# Toughening of Poly(lactic acid) and Thermoplastic Cassava Starch Reactive Blends Using Graphene Nanoplatelets

**DOI:** 10.3390/polym10010095

**Published:** 2018-01-19

**Authors:** Anibal Bher, Ilke Uysal Unalan, Rafael Auras, Maria Rubino, Carlos E. Schvezov

**Affiliations:** 1 School of Packaging, Michigan State University, East Lansing, MI 48824, USA; anibalbher@gmail.com (A.B.); iuysalunalan@gmail.com (I.U.U.); mariar@msu.edu (M.R.); 2 Instituto Sabato, UNSAM-CNEA, San Martin, Buenos Aires 1650, Argentina; 3 Instituto de Materiales de Misiones (IMAM), CONICET-UNaM, Posadas, Misiones 3300, Argentina; schvezov@gmail.com; 4 Department of Food Engineering, Faculty of Engineering, İzmir University of Economics, İzmir 35330, Turkey

**Keywords:** PLA, reactive blending, biobased films, graphene, nanoreinforcement

## Abstract

Poly(lactic acid) (PLA) was reactively blended with thermoplastic cassava starch (TPCS) and functionalized with commercial graphene (GRH) nanoplatelets in a twin-screw extruder, and films were produced by cast-film extrusion. Reactive compatibilization between PLA and TPCS phases was reached by introducing maleic anhydride and a peroxide radical during the reactive blending extrusion process. Films with improved elongation at break and toughness for neat PLA and PLA-*g*-TPCS reactive blends were obtained by an addition of GRH nanoplatelets. Toughness of the PLA-*g*-TPCS-GRH was improved by ~900% and ~500% when compared to neat PLA and PLA-*g*-TPCS, respectively. Crack bridging was established as the primary mechanism responsible for the improvement in the mechanical properties of PLA and PLA-*g*-TPCS in the presence of the nanofiller due to the high aspect ratio of GRH. Scanning electron microscopy images showed a non-uniform distribution of GRH nanoplatelets in the matrix. Transmittance of the reactive blend films decreased due to the TPCS phase. Values obtained for the reactive blends showed ~20% transmittance. PLA-GRH and PLA-*g*-TPCS-GRH showed a reduction of the oxygen permeability coefficient with respect to PLA of around 35% and 50%, respectively. Thermal properties, molecular structure, surface roughness, XRD pattern, electrical resistivity, and color of the films were also evaluated. Biobased and compostable reactive blend films of PLA-*g*-TPCS compounded with GRH nanoplatelets could be suitable for food packaging and agricultural applications.

## 1. Introduction

Poly(lactic acid) (PLA), an aliphatic polyester derived from lactic acid, is a biobased polymer that can be obtained from renewable sources, and in recent years has been used increasingly in food packaging and medical devices and in the agriculture, textile, and automotive industries [1]. PLA is recyclable, biodegradable, and compostable under industrial composting conditions, and it is considered to be an alternative to replace traditional fossil non-biodegradable polymers [2]. However, some drawbacks regarding PLA’s mechanical and barrier properties hinder its more widespread application in areas such as food packaging [1,3,4].

The blending of PLA with other polymers and the incorporation of nanofillers into the PLA matrix have been pursued to overcome some of the polymer’s shortcomings [5,6,7,8,9]. PLA has been blended with polyethylene glycol, ethylene vinyl alcohol, poly(butylene adipate-*co*-terephthalate), and other polymeric systems to improve crystallinity, biodegradability, and thermal and mechanical properties [10,11,12]. One of the main biopolymers used for reactive blending functionalization of PLA is a starch-based thermoplastic, in order to maintain PLA’s inherent compostability [13,14]. Starches obtained from crops such as corn, cassava, and sugar beets are cheap, biodegradable, and promote a lower environmental footprint [15]. Cassava starch is a good option for the production of thermoplastic cassava starch (TPCS) since cassava is inexpensive and is produced on a massive scale [16]. TPCS and modified TPCS offer acceptable properties as a PLA component for creating flexible packaging films [17,18].

Reactive blending is the best option to produce compatible PLA and TPCS blends [13,17,19,20]. The use of maleic anhydride in the presence of peroxide initiators, such as 2,5-bis(tert-butylperoxy)-2,5-dimethylhexane and dicumyl peroxide, has been widely reported as an efficient method to achieve an adequate compatibilization between the PLA and TPCS phases [13,17,20,21]. Films produced by reactive blending show a significant improvement in mechanical properties. However, the non-compatibilized or physical blends between these two biopolymers have poor mechanical properties and lower interfacial adhesion [17]. Although reactive blends of PLA-*g*-TPCS (i.e., PLA grafted to TPCS) present a good compatibilization of phases, the mechanical and barrier properties must be improved for the commercial production of films [21].

The use of clays (e.g., montmorillonite, halloysite), nanocellulose, metal nanoparticles (e.g., titanium dioxide, zinc oxide, silver), silica, and carbon nanotubes have been widely reported as reinforcement particles for some single or blended polymers [22,23,24].

Nanocomposites of PLA obtained using carbon-based nanofillers have been extensively reported with the aim of improving PLA’s properties by the incorporation of carbon-based materials with remarkable values of tensile strength, conductivity, thermal stability, etc. [25]. One of the most exciting novel nanomaterials used in polymers to improve properties is graphene. Graphene is produced from natural graphite and is considered a multifunctional material that can be introduced into polymers and composites. Low concentrations of graphene have been shown to improve polymer stiffness, electrical and thermal conductivity, and barrier properties due to its high surface area, high modulus, low density, etc. [26,27,28]. Graphene (GRH) nanoplatelets typically consist of a few graphene layers, with thicknesses between 1 and 10 nm, average particle diameters of 3 to 50 μm, and a high aspect ratio [29]. In comparison with the single layer, GRH nanoplatelets are low cost and can be used as a reinforcement nanomaterial with the potential for large-scale production [30].

The incorporation of GRH oxide into the matrix of PLA or starch composites results in mechanical and barrier properties improvements [31,32]. The diffusional pathway is influenced by the morphology of the nanosheets or nanoplatelets (size, aspect ratio, etc.) as well as the degree of exfoliation and orientation of the individual sheets or flakes in the polymer matrix [33]. However, the inclusion of GRH nanoplatelets in the matrix of PLA-*g*-TPCS blends has not yet been explored, and it could provide a unique opportunity for the production of a novel blend with unique properties. This work aimed to develop a reactive blend film of PLA-*g*-TPCS reinforced with a low level of GRH nanoplatelets (0.1 wt %), to understand the effect of adding this amount of GRH on PLA-*g*-TPCS reactive blends, and then to fully characterize the new material by molecular weight, microscopy, and mechanical, thermal, optical, electrical, and barrier properties.

## 2. Materials and Methods

### 2.1. Materials

Ingeo™ biopolymer 2003D poly (96% l-lactic acid) (PLA) was acquired from NatureWorks LLC (Minnetonka, MN, USA), with a weight (*M_w_*) and number (*M_n_*) average molecular weight of 2.2 ± 0.2 × 10^5^ Da and 1.2 ± 0.6 × 10^5^ Da, respectively. Cassava starch with amylose content of 25% ± 6% *wt*/*wt* and ~12% of moisture content was purchased from Aldema LLC (Cooperativa Agricola e Industrial San Alberto Ltda., Puerto Rico, Mnes, Argentina). Glycerol (>99.5%), maleic anhydride (MA), and 2,5-bis(tert-butylperoxy)-2,5-dimethylhexane (L101) were purchased from Sigma Aldrich (Milwaukee, WI, USA). Graphene nanoplatelets xGnP^®^ M-25, with an average particle diameter of 25 μm, a thickness of around 10 nm, and a surface area between 120 to 150 m^2^·g^−1^, were procured from XG Sciences Inc. (Lansing, MI, USA).

### 2.2. Production of Master Batch

PLA-*g*-MA was produced in a Century ZSK-30 twin-screw extruder (Century, Traverse City, MI, USA) with a composition of 2 wt % MA and 0.65 wt % L101, based on PLA weight. PLA, MA, and L101 were vigorously premixed in a bag before processing. The twin-screw extruder residence time for a rate of feed of 70 g·min^−1^ was ~3 min. The extruded mass was pelletized in a BT 25 pelletizer (Scheer Bay Co., Bay City, MI, USA), held in an oven at 50 °C for 3 h to remove residual water, and stored in a freezer at −15 °C until use. PLA-c (considered as control) was also produced, pelletized, and stored as previously described. TPCS was produced by mixing cassava starch and glycerol (in a resealable bag) in a proportion of 70/30 *wt*/*wt*, and holding for 12 h before use; TPCS was extruded and pelletized under similar conditions to those mentioned above. PLA-*g*-TPCS was produced by mixing PLA-*g*-MA with TPCS and using the same processing method as for PLA-*g*-MA. The proportion used of PLA-*g*-MA and TPCS was 70/30 *wt*/*wt*.

To produce the PLA-*g*-TPCS-GRH blend, pellets of PLA-*g*-TPCS were held in a vacuum oven overnight and were then premixed in a bag with 0.1 wt % of GRH nanoplatelets; the mixture was fed into the twin-screw extruder. For the PLA-GRH blend, neat PLA was premixed with 0.1 wt % of GRH and extruded using the twin-screw extruder, with an approximate residence time of 4 min. The extrusion was pelletized, held for 4 h at 50 °C in an oven, and stored in a freezer at –15 °C. Table 1 shows the processing conditions.

### 2.3. Production of Films

Master batches obtained from the twin-screw extruder were used to produce cast films in a single extruder (Randcastle Extrusion Systems, Inc., Cedar Grove, NJ, USA). Before the cast film process, all of the materials were conditioned overnight in an oven at 50 °C. The fabricated films were stored at −15 °C for further characterization. The processing conditions are presented in Table 2.

### 2.4. Molecular Weight

Weight average molecular weight (*M_w_*), number average molecular weight (*M_n_*), and polydispersity index (*PI*) of PLA-c and the fraction of PLA present in PLA-*g*-TPCS, PLA-GRH, and PLA-*g*-TPCS-GRH were determined by the method described in previous work [17,21] using a Waters gel permeation chromatograph (Waters Corp., Milford, MA, USA) equipped with a 1515 isocratic HPLC pump, a 717plus autosampler, four Styragel^®^ columns (HR1, HR2, HR3, and HR4), and a 2414 refractive index detector interface (Waters Corp.). Experiments were conducted in triplicate.

### 2.5. Scanning Electron Microscopy (SEM) and Atomic Force Microscopy (AFM)

SEM was used to investigate the morphology of the samples. Bar specimens of PLA-c, PLA-*g*-TPCS, PLA-GRH, and PLA-*g*-TPCS-GRH blends were produced by compression molding using pellets from the twin-screw extruder. Bars of PLA-*g*-TPCS and PLA-*g*-TPCS-GRH were immersed in liquid nitrogen for ~3 min, then fractured by hand, treated with hydrochloric acid (6 N) for 6 h to remove the TPCS phase, and air dried for 12 h in a fume hood [17]. Finally, the samples were mounted on aluminum stubs using carbon suspension cement (SPI Supplies, West Chester, PA, USA). Samples of films evaluated before and after tensile testing were also mounted on aluminum stubs with high vacuum carbon tabs (SPI Supplies) and coated with iridium at a thickness of ~2.7 nm. Samples were examined in a JEOL 6610LV (tungsten hairpin emitter) and a JEOL 7500F (field emission emitter) scanning electron microscope (JEOL Ltd., Tokyo, Japan) at various magnifications at 10 and 3.0 kV, respectively.

AFM was conducted using a Cypher™ atomic force microscope (Oxford Instruments Asylum Research, Inc., Santa Barbara, CA, USA) in the contact mode. Roughness parameters, calculated as the root mean square (*Rq*) and average roughness (*Ra*), were determined for each type of film and were calculated from the Htr mode image. Images were obtained in the Dfr mode. The film area for the determination of roughness was 900 μm^2^.

### 2.6. Profilometer

A profilometer NanoMap 500LS (AEP Technology, Santa Clara, CA, USA) was also used for roughness determination. Films were attached to a microscope slide for measuring, and scans were conducted at 50 μm·s^−1^ with a sample frequency of 100 pts·s^−1^, data resolution of 5 μm, 6000 number of points per scan, and a contact force of 5.4 mg. The scan area was 9 × 10^6^ μm^2^. Values of *Ra* (nm) and peak-to-peak (nm) were determined from every measurement. A minimum of two samples was scanned for each film.

### 2.7. Tensile Properties

Tensile strength, elongation at break, and Young’s modulus were evaluated using an Instron^®^ Universal Machine 5565 (Instron, Norwood, MA, USA) according to ASTM D882-12 [34]. Film samples were cut into 2.54 cm × 20 cm strips and conditioned for 48 h in an environmental chamber (Environmental Growth Chambers, Chagrin Falls, OH, USA) at 23 °C and 50% relative humidity. PLA-c was tested with an initial grip and a rate grip separation of 125 mm and 12.5 mm·min^−1^, respectively. All of the other samples were tested with an initial grip and a rate grip separation of 100 mm and 50 mm·min^−1^, respectively. Five samples were evaluated for each formulation. Film thickness was determined by averaging five measurements for every specimen using a digital micrometer (Testing Machines Inc., Ronkonkoma, NY, USA).

### 2.8. Thermal Properties

Differential scanning calorimetry (DSC) of the films was carried out with a Q100 differential scanning calorimeter (TA Instruments, New Castle, DE, USA) equipped with a mechanical cooling system. Specimens between 5 and 10 mg were cut, weighed, and sealed in an aluminum pan. Samples were first cooled from room temperature to −50 °C, heated from −50 °C to 200 °C (first heating cycle), underwent an isothermal for 1 min at 200 °C, again cooled until −50 °C, and finally heated to 200 °C (second heating cycle). The sample purge flow rate was 70 mL·min^−1^ of nitrogen. Glass transition temperature (*T_g_*), cold crystallization temperature (*T_cc_*), melting temperature (*T_m_*), and degree of crystallinity (*X_c_*) of PLA and blends were determined using the TA Universal Analysis 2000 software, version 4.5A (TA Instruments, New Castle, DE, USA). The *X_c_* of PLA was calculated from an equation modified from Detyothin et al. [17]:

(1) $$X_{c}=\frac{\Delta H_{m}-\Delta H_{c}}{\Delta H_{f}\times \left(1-\alpha \right)}$$

where $\Delta H_{m}$ and $\Delta H_{c}$ are the enthalpies of melting and crystallization, respectively; $\Delta H_{f}$ is the enthalpy of fusion of a pure crystalline PLA with a value of 93 J·g^−1^ [35]; and α is the sum of the weight fraction of TPCS, MA, and GRH in the final blends. The values of *X_c_* are reported from the first and second heating run. Samples were run in triplicate.

Thermogravimetric analysis (TGA) of the films was conducted in a Q50 thermal gravimetric analyzer (TA Instruments) under a nitrogen atmosphere with a flow rate of 60 mL·min^−1^. The method used was a ramp of 10 °C·min^−1^ from room temperature until reaching 600 °C in an aluminum pan. Between 5 and 10 mg of sample was used for every run. Samples were run in triplicate.

Dynamic mechanical analysis (DMA) was conducted using an RSA-G2 analyzer (TA Instruments). Film samples (64 mm × 12 mm) were conditioned at 50% relative humidity and 23 °C for 48 h in an environmental chamber. Five specimens were evaluated for each type of blend. The storage modulus (*Eʹ*), loss modulus (*Eʺ*), and tan delta were evaluated. A loading gap of 15 mm for a rectangular geometry was used. A tension axial force of 400 g with a sensitivity of 10 g was used, with an oscillation temperature ramp of 3 °C·min^−1^ from 25 to 100 °C with a frequency of 1.0 Hz and a strain of 2%. Five specimens of each type of blend were assessed.

### 2.9. UV-Visible Spectroscopy

Transmittance of the films was evaluated with a Lambda 25 UV/Vis spectrophotometer (PerkinElmer, Waltham, MA, USA) in a wavelength range of 190–880 cm^−1^ using one cycle, with a scan speed of 480 nm·min^−1^.

### 2.10. Barrier Properties to Oxygen and Water Vapor

Oxygen and water vapor barrier performance of film samples were measured with Ox-tran^®^ model 2/21 and Permatran^®^ model 3/33 testing instruments (Mocon, Minneapolis, MN, USA), respectively. Samples were mounted in masks of aluminum foil with an exposed area of 3.14 cm^2^. Test conditions were 30% relative humidity (RH) and 23 °C for oxygen and water vapor. The oxygen test was conducted with air.

### 2.11. Statistical Analysis

Statistical analysis was conducted with MINITAB™ software (State College Park, PA, USA). ANOVA and Tukey’s test were used to evaluate the comparison of means at *p* ≤ 0.05.

## 3. Results and Discussion

Reactive blends of PLA and TPCS compatibilized with MA using L101 as peroxide initiator were produced and characterized. GRH nanoplatelets were introduced with the objective of increasing the toughening of the reactive blends. Subsequently, cast films were produced and characterized. PLA-c and PLA-GRH were produced as reference films, and their properties are also reported.

### 3.1. Molecular Weight

The *M_n_*, *M_w_*, and *PI* values of the PLA films are reported in Table 3. The *M_n_* and *M_w_* for the fraction of PLA measured decreased significantly for the reactive blend of PLA-*g*-TPCS with respect to PLA-c. The overall reduction of the PLA-*g*-TPCS is related to several factors: (1) the use of MA and the peroxide initiator; (2) the presence of water and glycerol on TPCS; (3) the use of a two-step processing method (twin-screw extruder for the production of blends and single extruder for the production of films); and (4) the thermal conditions associated with the process.

The degradation of PLA in the reactive blends can be associated with the dominant side reaction occurring in the maleated PLA obtained using a free radical grafting initiator, such as L101, and a compatibilizer, such as MA [19].

The presence of water due to the incorporation of TPCS for the production of the reactive blend is a reasonable factor affecting the reduction of the *M_n_* and *M_w_* of PLA [36]. Hydrolytic degradation is a well-known degradation mechanism of PLA due to the presence of moisture. The hydrolysis of PLA begins by the diffusion of water molecules into the amorphous portion of PLA, resulting in the cleavage of the ester bonds [37]. Thermal degradation of PLA during processing is also related to the hydrolysis of residual water, main-chain scission, and intra and intermolecular transesterification. A reduction of *M_n_* for PLA by hydrolysis was reported in the presence of water, even at low temperatures [38]. The presence of low molecular weight additives, such as glycerol, has a negative impact on the molecular weight of PLA due to thermal degradation or the hydrolysis of polyester chains.

The production of films for characterization required a two-step processing method. In this case, the use of different temperatures and shear and mixing conditions allow for a reduction of molecular weight. Taubner and Shishoo reported a reduction of *M_n_* for PLA during melt extrusion using a double-screw extruder influenced by the processing temperature and residence time [39].

In the case of PLA-GRH, the values of *M_n_* and *M_w_* were similar to those of PLA-c, showing that the addition of GRH nanoplatelets did not affect the PLA’s structure. In the case of the final reactive blend, PLA-*g*-TPCS-GRH, this resulted in a ~30% decrease of *M_n_* and *M_w_* with respect to PLA due to the dual processing steps of the composite blends: first, to produce the reactive blend and then due to the addition of the GRH nanoplatelets and reprocessing. This reduction is associated with thermal conditions inherent with the processing in the twin-screw and single extruders. Nonetheless, the values of *PI* showed a narrow distribution of the molecular weight for all the samples: *PI* ≤ 2.

### 3.2. Morphology of the Films

**Figure 1:**
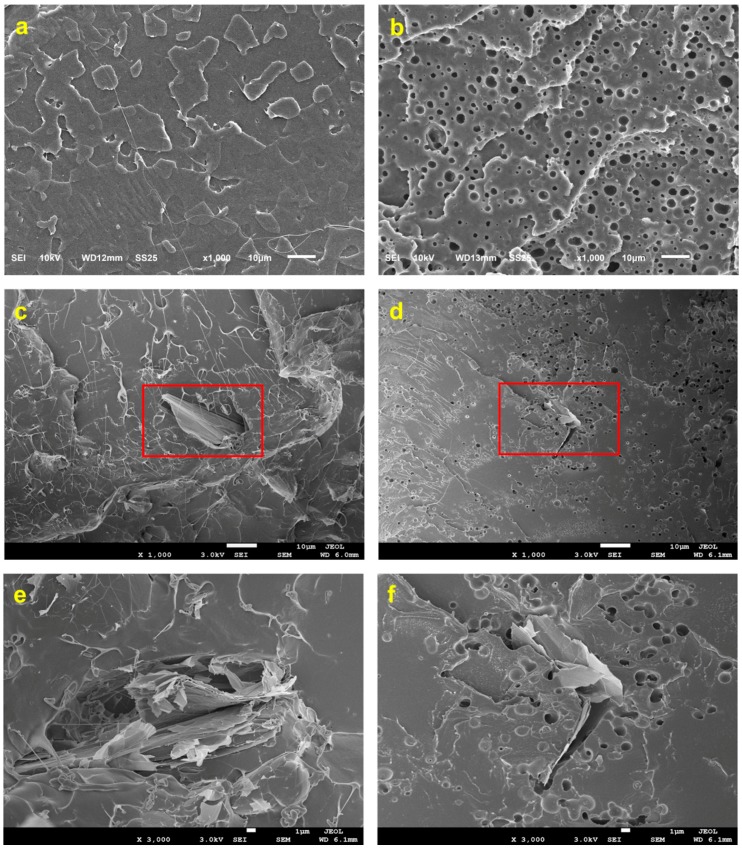
SEM images of film samples showing the polymer domains and distribution of GRH nanoplatelets: (**a**) PLA-c; (**b**) PLA-*g*-TPCS; (**c**) PLA-GRH (×1000); (**d**) PLA-*g*-TPCS-GRH (×1000); (**e**) PLA-GRH (×3000); (**f**) PLA-*g*-TPCS-GRH (×3000).

SEM images of the PLA blends were obtained to understand the grade of compatibilization between PLA and TPCS and the incorporation of GRH nanoplatelets. An optimal distribution of GRH nanoplatelets should play an important role in enhancing the barrier and mechanical properties of nanocomposite blends. The morphology of the fracture surface for the evaluated blends is shown in Figure 1. Figure 1a shows the surface of PLA-c. Figure 1b shows the reactive blend of PLA-*g*-TPCS after removing the TPCS phase; good compatibilization between PLA and TPCS was achieved in the reactive blend by the incorporation of MA and L101. Similar results for these reactive blends were reported by Detyothin et al. [17]. The proportion of cavities observed due to the removal of TPCS is in accordance with the proportion used for the production of the reactive blend. The compatibilization achieved allowed for good homogenization during the processing step using a twin-screw extruder. The incorporation of GRH nanoplatelets into neat PLA is presented in Figure 1c, where a small distribution of GRH nanoplatelets in the PLA matrix is in accordance with the low load of GRH used in the production (0.1 wt %). Figure 1d shows the presence and distribution of GRH nanoplatelets in the matrix of the compatibilized blend obtained with PLA and TPCS. By comparing Figure 1c,d, it appears that the GRH nanoplatelets are mostly located as flakes in the interface of PLA and TPCS. Figure S1 (supporting information available online) shows the graphene nanoplatelets powder used as a reinforcement and the formation of agglomerates. The TPCS domain was removed in Figure 1d, and it is apparent that the GRH nanoplatelets are embedded in the cavities surrounded by the PLA matrix. This observation may help explain the mechanical property results whereby the GRH nanoplatelets increased the tensile strength of PLA-GRH with respect to neat PLA but also increased the elongation at break of the reactive blend produced with TPCS.

Figure 1e,f reveal further details of how the GRH nanoplatelets are inserted in both the PLA matrix and the PLA-*g*-TPCS-GRH. An uneven distribution of nanoplatelets (but good compatibilization and adhesion) in the matrix is evident. Figure 1e,f show the clustering or agglomeration of several GRH nanoplatelets, which could affect the barrier and mechanical properties. The presence of flakes of GRH in the matrix of PLA-c and PLA-*g*-TPCS exhibiting a surface structure with cracks was also reported by Gao et al. [40] working with nanocomposites of PLA and GRH nanoplatelets at concentrations between 5 and 15 wt %. Pinto et al. also reported deficient exfoliation of GRH nanoplatelets with aggregations of 5 to 10 sheets, as observed by SEM, and overlapping of nanoplatelets as observed by optical microscopy [26]. How the distribution of GRH nanoplatelets in the polymer matrix affected the mechanical properties of the films is described in the next section.

**Figure 2:**
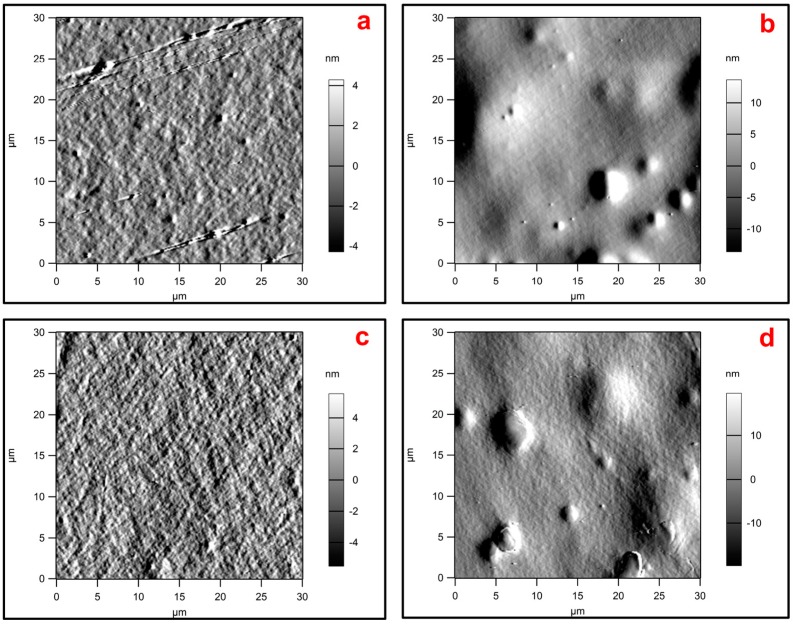
AFM images of films: (**a**) PLA-c; (**b**) PLA-*g*-TPCS; (**c**) PLA-GRH; (**d**) PLA-*g*-TPCS-GRH.

Table 4 and Figure 2 present the surface roughness of the PLA films as evaluated by AFM. The PLA-*g*-TPCS and PLA-*g*-TPCS-GRH films had higher values of surface roughness in comparison with the PLA-c and PLA-GRH films. This greater roughness could be attributed to the presence of the starch matrix in both reactive blends. Figure 2 shows a smooth surface for PLA-c and PLA-GRH. Roughness values obtained by profilometry are larger than those obtained by AFM due to the larger scan area; however, the values follow the same trend (i.e., roughness for PLA-c and PLA-GRH are lower than for PLA-*g*-TPCS and PLA-*g*-TPCS-GRH.).

Figure 1a,b allow for a comparison of the difference observed in roughness by AFM due to the presence of the TPCS phase. An increase of roughness could be attributed to irregular shapes of starch granules as can be observed for the cavities where these granules were immersed. The creation of a topography with different values of roughness could be due to the presence of two different phases that were compatibilized.

The presence of GRH nanoplatelets could not be significantly affecting the roughness of the PLA films due to the low load used for film production, and its effect is masked in the reactive blends due to the effect of the TPCS phase. Pinto et al. evaluated the topography of PLA with GRH nanoplatelets as a nano-based material and reported an increase in roughness with a higher concentration of GRH nanoplatelets (0.4 wt %) [41].

### 3.3. Tensile Properties

**Figure 3:**
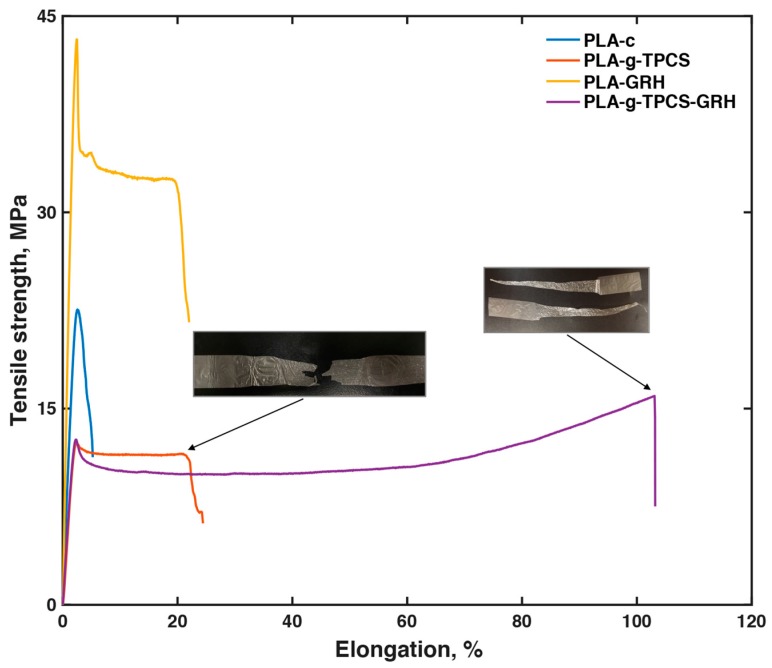
Tensile strength vs. elongation at break for films produced by twin-screw extrusion–cast-film extrusion.

An analysis of the tensile testing of the PLA films, evaluated in the machine direction, reveals a characteristic brittle behavior for PLA-c, with tensile strength values of ~25 MPa and elongation at break values around 9% (Table 5 and Figure 3). However, the introduction of GRH nanoplatelets to the PLA matrix resulted in improvements in both tensile strength and elongation at break of ~75% and 130%, respectively. Others have reported similar values of tensile strength when GRH nanoplatelets were used as a nanofiller for PLA [42]. Chieng et al. [43] reported tensile strength values of ~60 MPa for PLA with 0.3 wt % of GRH. Valapa et al. [27], using expandable graphite as reinforcement, found similar increments for elongation at break of PLA, but the increment for tensile strength was small with the same load (0.1 wt %).

The Young’s modulus increased by ~100% with respect to neat PLA, indicating the stiffness behavior of the incorporated nanofiller. Reactive blending using MA and L101 to obtain better compatibilization between PLA and TPCS resulted in a blend with good elongation at break (~25%) but a ~50% reduction in tensile strength with respect to PLA-c. Similar values have been previously reported for these blends [17]. The addition of GRH nanoplatelets to the reactive blend of PLA-*g*-TPCS resulted in an important improvement in the elongation at break (values larger than 100%), showing an increment of ~300% with respect to PLA-*g*-TPCS. Values of toughness showed a significant improvement for PLA-*g*-TPCS-GRH, around ~900% with respect to PLA-c. Figure 3 insets are images of the films (PLA-*g*-TPCS and PLA-*g*-TPCS-GRH) after the tensile testing. The nanofiller incorporated did not improve the tensile strength of the reactive blend.

Figure 3 indicates that at least two mechanisms were acting when PLA-*g*-TPCS-GRH films were tested: a well-identified yield point and a strain hardening behavior. In Figure 1, the presence of gaps between the GRH nanoplatelets and the polymer matrix in the fractured surface of PLA-*g*-TPCS-GRH could be identified. Furthermore, Figure S2 shows the SEM images of the films after the tensile test. The cavities generated in the PLA-*g*-TPCS polymer matrix during the tensile test can be observed, which could be one of the main reasons for the improvement in the elongation at break (Figure S2b,e). Due to the incorporation of GRH nanoplatelets, when the material is under tension, the fractures created in the surface of the polymer matrix could find a free propagation path or a flake of GRH nanoplatelets. Since the GRH nanoplatelets are stiff materials, in the second case the fracture is forced to find an alternative path that continues with the propagation of the fracture and breaks the material during plastic deformation. Thus, an increase of the deformation energy and toughening is observed due to the addition of GRH, which is finally translated into high values of elongation at break [27,44].

The presence of GRH nanoplatelets, even at a low concentration, is enough to create a crack-bridging mechanism during tension [45]. This mechanism could increase the fracture toughness of a nanocomposite, and its efficiency is important when the nanofiller has a high value of aspect ratio [44]. One of the most significant properties of GRH nanoplatelets is their high aspect ratio. Since the adhesion of the GRH nanoplatelets to the PLA matrix is strong—as shown in the SEM images, Figure 1d and Figure S2f,l—and the nanoplatelets can act as a bridge between two fracture surfaces, they are avoiding or delaying the pullout and thus increasing the fracture energy, as was also demonstrated for other fillers [44,45,46].

On the other hand, it has been reported that when PLA was loaded with GRH nanoplatelets above 0.3 wt %, the elongation at break decreased and that was attributed to the large load of GRH nanoplatelets restricting the mobility of the polymer chains [42,47]. A high load of GRH nanoplatelets in a polymer matrix allows for the restack of nanosheets due to a Van der Waals force. As a consequence, under tension the primary effect will be the slippage of the graphene nanosheets with lower values of tensile strength [47].

Gao et al. [40] also concluded that the incorporation of graphene nanoplatelets (~15 nm) at a higher load (e.g., 5 to 10%) reduced the mobility of the PLA chains in the composite and increased its brittle behavior, which could be attributed to some aggregation of nanoplatelets due to the high concentration.

### 3.4. Thermal Properties

**Figure 4:**
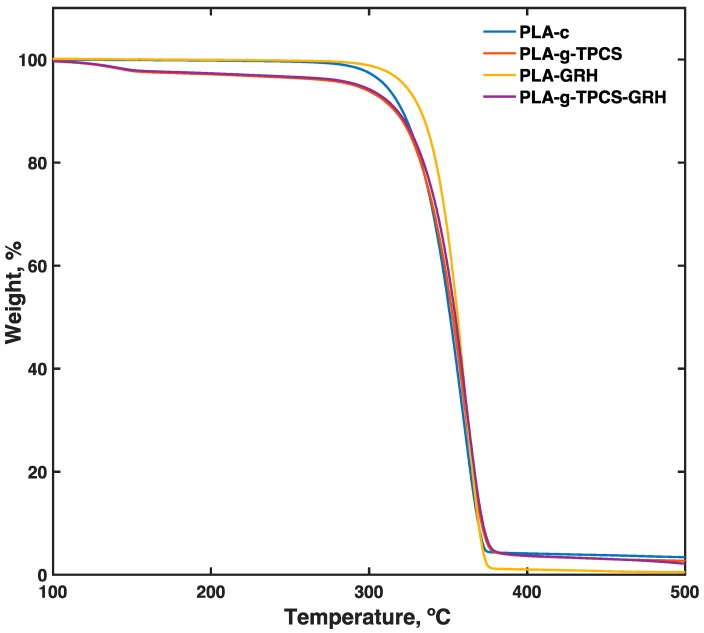
Thermogravimetric analysis (TGA) thermograms of the tested samples produced by twin-screw extrusion–cast-film extrusion.

Figure 4 shows the TGA results for the evaluated samples. No significant difference in onset decomposition temperature was observed among neat PLA, PLA-*g*-TPCS, and samples with the GRH nanoplatelets incorporated into these matrixes; all samples had an onset temperature between 310–320 °C. An early change (before the onset temperature) was depicted, mainly due to the presence of the TPCS phase and the presence of glycerol. The incorporation of a nanofiller has been shown to enhance composite thermal stability; this was evident for PLA-GRH, but this was not evident for the addition of GRH nanoplatelets at low load (i.e., 0.1 wt %) in the PLA-*g*-TPCS blends. Chieng et al. [42], working with a plasticized PLA reinforced with GRH nanoplatelets, reported a similar value for the onset decomposition temperature. The addition of a load of GRH nanofiller above 0.5 wt % into the PLA matrix was shown to significantly improve thermal stability [42]. The incorporation of GRH nanoplatelets at higher loads could work as a heat barrier, shifting the decomposition of the composite to higher temperatures, and also, due to its high aspect ratio, create a barrier for volatile degradation products present in the nanocomposite [48]. Similar values of onset decomposition temperature (*T_onset_*) and maximum decomposition temperature (*T_dmax_*) were observed for PLA and the reactive blends with and without the addition of GRH nanoplatelets (Table 6). Residual values observed are due to the decomposition of organic matter of TPCS and the formation of ash [49]. A lower residual was observed for PLA-GRH. This could be associated with the fact that GRH nanoplatelets are thermally conductive, and thus could improve the loss of residual mass of this blend at high temperature. Additionally, there is no presence of ash derived from the cassava starch.

**Figure 5:**
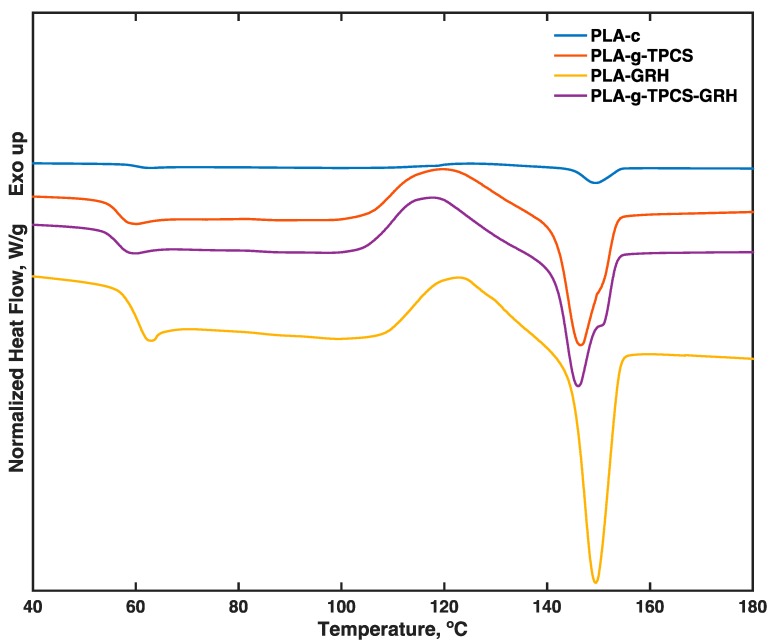
DSC thermograms of the second heating cycle of the tested films; samples were produced by twin-screw extrusion followed by cast-film extrusion.

The DSC results for the second heating cycle are presented in Table 6 and Figure 5. With respect to PLA-c, reductions in *T_g_* of ~10 °C for the reactive blend PLA-*g*-TPCS and ~5 °C for the reactive blend with GRH nanoplatelets were observed. However, PLA-GRH had a similar *T_g_* to PLA-c. Valapa et al. [27] also reported that a low content of expandable graphite in PLA composites did not affect the *T_g_*, concluding that an incorporation of a low amount of reinforcement does not affect the mobility and reduction of PLA chains. On the other hand, lower *T_g_* values have been reported for plasticized PLA nanoreinforced with GRH nanoplatelets [42], although the loads were higher (0.3 to 1.0 wt %). A similar reduction in *T_g_* has been reported due to the presence of a TPCS phase and the use of MA and L101 [17]. The incorporation of GRH nanoplatelets in the PLA-*g*-TPCS increased its *T_g_* compared with the unreinforced blends; this finding could be correlated with the tensile test results, whereby the GRH nanoplatelets acted as a reinforcement of the blends, reducing PLA chain mobility. The reactive blends had lower *T_m_* values in comparison to PLA-c, and this decline could be associated with the reduction in *M_n_*, which has a larger effect on *T_m_* than on *T_g_*. PLA-GRH exhibited a small decrease in *T_m_*.

The values of crystallization, as determined by the second heating cycle, remained stable for all of the blends, which are mostly amorphous. The presence of GRH nanoplatelets did not modify the crystallization behavior of PLA, as observed in Figure S3. A small crystallization peak is shown at 16.4° and assigned to the plane (200)/(110) of the α-crystal of PLA, confirming the presence of an ordered region due to the presence of the nanofiller [32]. However, the blending with TPCS disrupts the crystallization process, creating two types of crystal forms (α and α’) as previously reported [50], which are difficult to crystallize. The XRD patterns (Figure S3) exhibited a fully amorphous behavior for all of the films produced, with a crystallization peak for PLA-GRH at 16.4° and a peak showing the presence of GRH nanoplatelets in PLA-GRH and PLA-*g*-TPCS-GRH at 26.5° (corresponding with the *d*002 spacing of graphite). The presence of this peak at 26.5° confirms that a high percentage of GRH nanoplatelets were intercalated in the polymer matrix. The thermal parameters obtained from the first heating cycle of the DSC (Table S1) showed that the crystallinity of all of the samples was mostly amorphous.

**Figure 6:**
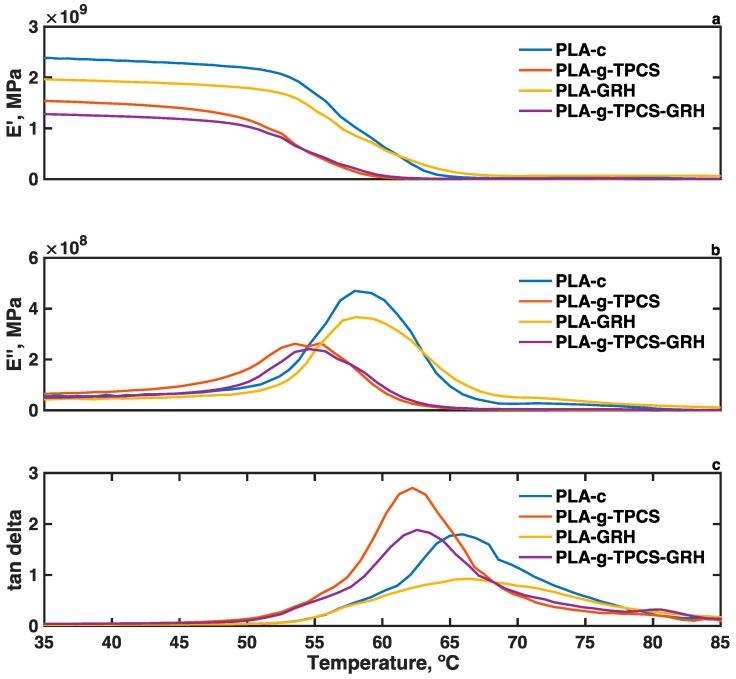
Dynamic mechanical analysis (DMA) thermograms of films produced by twin-screw extrusion–cast-film extrusion: (**a**) *Eʹ*, storage modulus; (**b**) *Eʺ*, loss modulus; and (**c**) tan delta.

Figure 6a shows that the storage moduli, *Eʹ*, is reduced in the range of 35 to 60 °C due to the addition of TPCS phase concerning the PLA-c, indicating a reduction of the elastic region for PLA-*g*-TPCS. The reduction observed for *E’* upon the incorporation of GRH in PLA is due to the increase in toughening and the more plastic behavior of PLA-GRH. The *E*’ represents the stiffness of the viscoelasticity of PLA and is proportional to the energy stored during the loading cycle. Since the addition of GRH improves the Young’s Modulus of PLA-GRH samples and also the toughness of the samples through the crack-bridging mechanism as previously described, the increase of the Young’s Modulus is due to the addition of the GRH nanoplatelets (the modulus is around 1 TPa for GRH), and the simultaneous reduction of *E’* is an indication of a less elastic and more plastic deformation with the temperature of the PLA-GRH sample. This incorporation of GRH into PLA-*g*-TPCS has less effect on the reduction of *E’* due to the presence of the plastic TPCS phase. With the increment of temperature after 60 °C, a slow decrease is observed for *Eʹ* until it reaches the glass transition region where the drop of *E’* is important. Above the glass transition region, the values for *Eʹ* are similar for all of the evaluated samples. An important reduction of *Eʺ* is observed for the reactive blends with respect to PLA. Thus, the reactive blends showed a tough behavior with less energy dissipated during deformation.

The *T_g_* values have been reported to be higher when estimated by DMA than by DSC [51]. Nevertheless, the *T_g_* trend observed in the DMA was the same as that observed in the DSC analysis, with similar values for PLA-c and PLA-GRH and a shift to lower temperatures for PLA-*g*-TPCS and PLA-*g*-TPCS-GRH. This shift is associated with the enhanced chain mobility of PLA due to the TPCS phase and the plasticizing effect achieved by reactive compatibilization. The plasticization effect could be described as the softening of the blend due to the presence of the glycerol. However, the decrease of the tan delta peak with the addition of GRH indicates that the polymer chains during the transition region are less restricted by the GRH nanoplatelets. Others have suggested that the reinforcement due to the addition of GRH nanoplatelets leads to a restriction of the chain mobility of the polymer [52]. A similar reduction of tan delta peaks was reported by Jonoobi et al. [53] when using cellulose nanofibers to reinforce PLA.

### 3.5. Optical Properties of Films

**Figure 7:**
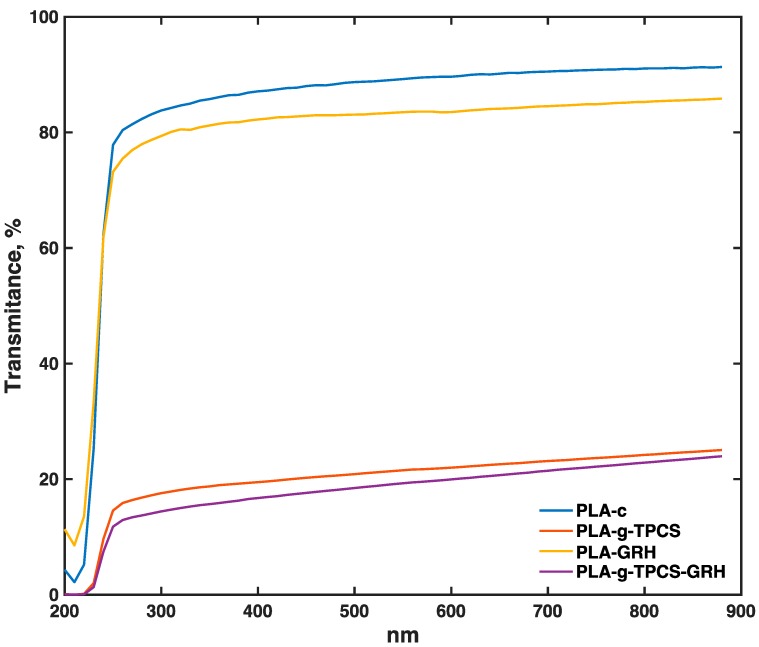
Transmittance (%) of film samples produced by twin-screw extrusion–cast-film extrusion.

Transmittance of the characterized films is presented in Figure 7. The films can be divided into two groups. In the first group, comprising PLA-c and PLA-GRH, a ~10% reduction in transmittance between 250 and 880 nm was observed between the films due to the presence of GRH nanoplatelets. The films in the second group, PLA-*g*-TPCS and PLA-*g*-TPCS-GRH, had similar transmittance values, but these values were ~75% lower with respect to the first group. This phenomenon is mainly due to the presence of TPCS in the matrix of PLA-*g*-TPCS and PLA-*g*-TPCS-GRH, as can be observed in Figure S4, and which was discussed previously in terms of the increase of roughness in these films, as determined by AFM. A full characterization of the color and opacity of the film samples is provided in Table S2. The incorporation of 0.1% of nanofiller into PLA has shown similar characteristics as those reflected in this work [27].

It is also important to note that the presence of GRH in PLA-GRH and PLA-*g*-TPCS-GRH plays an important role in the electrical properties of these materials. The values of electrical resistivity for the films are provided in Table S3, and indicate that sample conductivity increases with the addition of very low amounts of GRH nanoplatelets. Higher values of conductivity were reported by Gao et al. [40] working with higher loads of GRH nanoplatelets (5–15%).

### 3.6. Barrier Properties to Oxygen and Water Vapor

Table 7 depicts the oxygen and water vapor permeability coefficients for the films tested under conditions of 30% RH and 23 °C. The oxygen permeability coefficients were lower in PLA films with GRH nanoplatelets, with a further improvement for the reactive blend with GRH nanoplatelets. However, no reduction was observed due to the presence of TPCS. The water vapor permeability did not decline due to the addition of TPCS, perhaps due to the low RH testing condition. Furthermore, the water vapor permeability coefficient for the reactive blend did not decline due to the addition of the nanofiller. The presence of GRH nanoplatelets in the films tends to reduce the permeability to oxygen; nevertheless, due to the low load used, this improvement was not reflected in an important reduction in permeability coefficient value. The SEM images (Figure 1) showed the formation of flakes with several GRH nanoplatelets and poor dispersion. This could be one reason for the lack of oxygen barrier improvement. Research done in this field has demonstrated that the presence of graphene (nanosheets or nanoplatelets) improves the barrier properties of PLA and PLA blends. Huang et al. [54] presented three main factors affecting the “tortuosity” for the penetration and diffusion of molecules: (1) the volume fraction of nanoplatelets; (2) the morphology of the nanoplatelets (e.g., exfoliation, dispersion, and orientation perpendicular with respect to the direction of diffusion); and (3) the aspect ratio. Exfoliation of GRH nanosheets was reported to improve the barrier permeability to oxygen on PLA with a load of 0.1%, but had a negative effect with a load of 0.5%, which could be associated with the formation of aggregates at high loadings [27]. The presence of GRH nanosheets in PLA/starch blends was reported by Wu et al. [32], who demonstrated an important improvement in oxygen permeability for blends with ~30% starch and that were reinforced with 0.1% graphene nanosheets.

## 4. Conclusions

Reactive functionalization of GRH nanoplatelets in the compatibilized matrix of PLA and thermoplastic cassava starch (TPCS) resulted in a significant improvement in elongation at break. The presence of GRH nanoplatelets in the PLA-*g*-TPCS reactive blend increased their toughness, with an improvement of ~900% and ~500% when compared to neat PLA and PLA-*g*-TPCS, respectively. GRH nanoplatelets in the reactive blend of PLA-*g*-TPCS were mainly located in the interface and were agglomerated in several flakes. The mechanism of crack bridging was identified as being responsible for the improvement in elongation at break for the reactive blends and the increase in the Young’s modulus for PLA with the addition of GRH nanoplatelets. The use of two-step extrusion resulted in a large reduction, and hence a negative effect, on the molecular weight of PLA. Using a one-step processing condition could significantly improve the *M_w_* for PLA-*g*-TPCS and PLA-*g*-TPCS-GRH. Production of the reactive blends by using a twin-screw extruder and a sheet film die adapted at the end of the extruder should be explored to scale this blend. Surface roughness was greater for the reactive blends with and without GRH. Transmittance decreased for the reactive blends with and without GRH in the UV-visible range, mainly due to the presence of the starch phase. Nevertheless, the reactive blends are still acceptable for see-through applications. Oxygen barrier properties improved with the addition of GRH nanoplatelets to PLA-c and PLA-*g*-TPCS. However, water barrier properties did not improve with the addition of GRH nanoplatelets; one of the main reasons could be the scarce exfoliation and orientation achieved. Moreover, the formation of flakes of GRH nanoplatelets in the polymer matrix could be diminishing the ability to create a tortuous path. Future work will focus on improving the barrier properties of these reactive blends with GRH nanoplatelets as a nanofiller reinforcement.

## Figures and Tables

**Table 1:** Conditions of processing of master batches in the twin-screw extruder.

Films	Temperature Profile from the Feeder to the Die (°C)	Screw Speed (RPM)
PLA-c	140/150/160/160/160/170/170/170/170/160	120
TPCS	25/100/105/110/115/120/120/120/115/115	125
PLA-*g*-MA	140/150/160/160/160/170/170/170/170/160	120
PLA-*g*-TPCS	140/150/160/160/160/170/170/170/170/160	120
PLA-GRH	140/150/160/160/160/170/170/170/170/160	100
PLA-*g*-TPCS-GRH	140/150/160/160/160/170/170/170/170/160	100

RPM: revolutions per minute; PLA: Poly(lactic acid); TPCS: thermoplastic cassava starch; MA: maleic anhydride; GRH: graphene.

**Table 2:** Processing conditions of the cast films.

Films	Temperature Profile from the Feeder to the Die (°C)	Screw-Nip Roller-Winding Roller Speed (RPM)
PLA-c	140/150/160/160/160/170/160	30-50-12
PLA-*g*-TPCS	140/150/160/160/160/170/160	30-50-12
PLA-GRH	140/150/160/160/160/170/160	30-50-12
PLA-*g*-TPCS-GRH	140/150/160/160/160/170/140	30-50-12

**Table 3:** *M_n_*, *M_w_*, and *PI* of PLA-c and the PLA portion of the various films produced.

Films	*M_n_*	*M_w_*	*PI*
PLA-c	103.5 ± 0.1 ^a^	201.9 ± 0.4 ^a^	1.9 ± 0.1 ^a^
PLA-*g*-TPCS	71.6 ± 10.5 ^b^	147.6 ± 2.5 ^b^	2.0 ± 0.3 ^a^
PLA-GRH	98.8 ± 3.1 ^a^	200.5 ± 0.7 ^a^	2.0 ± 0.1 ^a^
PLA-*g*-TPCS-GRH	71.0 ± 1.3 ^b^	138.1 ± 1.3 ^c^	1.9 ± 0.1 ^a^

Note: Within columns, values followed by a different letter are significantly different at *p* ≤ 0.05 (Tukey’s test). *PI*: polydispersity index.

**Table 4:** Roughness of PLA films as measured by AFM and profilometry.

	AFM		Profilometry	
Films	*Rq* (nm)	*Ra* (nm)	*Ra* (nm)	Peak-to-Peak (nm)
PLA-c	9.0 ± 2.1 ^a^	6.3 ± 1.3 ^a^	1046 ± 572 ^a^	5140 ± 2023 ^a^
PLA-*g*-TPCS	183.5 ± 0.3 ^b^	143.6 ± 5.4 ^b^	35,167 ± 12,176 ^b^	126,667 ± 23,283 ^b^
PLA-GRH	12.5 ± 2.3 ^a^	9.8 ± 1.7 ^a^	2811 ± 991 ^a^	13,986 ± 4577 ^a^
PLA-*g*-TPCS-GRH	141.8 ± 33.5 ^b^	104.4 ± 37.4 ^b^	17,100 ± 7353 ^b^	66,350 ± 19,728 ^c^

Note: Within columns, values followed by a different letter are significantly different at *p* ≤ 0.05 (Tukey’s test).

**Table 5:** Tensile properties of films produced by cast-film extrusion.

Films	Thickness, μm	Tensile Strength, MPa	Modulus, GPa	Elongation at Break, %	Toughness, MJ·m^−3^
PLA-c	24.2 ± 3.5 ^a^	24.7 ± 0.7 ^a^	1.2 ± 0.1 ^a^	8.9 ± 5.1 ^a^	1.3 ± 0.7 ^a^
PLA-*g*-TPCS	46.0 ± 5.6 ^b^	11.0 ± 1.5 ^b^	0.7 ± 0.1 ^b^	23.9 ± 2.1 ^b^	2.3 ± 0.5 ^a^
PLA-GRH	20.8 ± 2.9 ^a^	43.2 ± 1.5 ^c^	2.2 ± 0.3 ^c^	21.2 ± 5.7 ^b^	5.3 ± 1.1 ^b^
PLA-*g*-TPCS-GRH	50.6 ± 8.4 ^b^	13.7 ± 1.1 ^b^	0.8 ± 0.1 ^b^	103.4 ± 2.7 ^c^	13.0 ± 1.4 ^c^

Note: Within columns, values followed by a different letter are significantly different at *p* ≤ 0.05 (Tukey’s test).

**Table 6:** *T_g_*, *T_cc_*, *T_m_*, and *X_c_* from the second heating cycle of differential scanning calorimetry (DSC), and *T_onset_* and *T_dmax_* from TGA.

Films	*T_g_*, °C	*T_cc_*, °C	*T_m_*, °C	*X_c_*, %	*T_onset_*, °C	*T_dmax_*, °C
PLA	61.2 ± 0.7 ^a^	93.2 ± 4.2 ^a^	150.6 ± 0.2 ^a^	3.1 ± 0.6 ^a^	318.9 ± 8.4 ^a^	360.8 ± 1.7 ^a^
PLA-*g*-TPCS	52.3 ± 0.5 ^b^	107.4 ± 0.5 ^b^	143.4 ± 0.3 ^b^	1.2 ± 0.6 ^a^	310.4 ± 6.3 ^a^	359.3 ± 0.4 ^a^
PLA-GRH	61.0 ± 0.0 ^a^	124.0 ± 0.8 ^c^	149.5 ± 0.2 ^c^	2.2 ± 0.6 ^a^	320.1 ± 5.7 ^a^	360.7 ± 0.8 ^a^
PLA-*g*-TPCS-GRH	57.3 ± 0.1 ^c^	118.8 ± 1.3 ^c^	146.3 ± 0.2^d^	2.0 ± 0.9 ^a^	309.0 ± 2.7 ^a^	359.9 ± 0.4 ^a^

Note: Within columns, values followed by a different letter are significantly different at *p* ≤ 0.05 (Tukey’s test).

**Table 7:** Barrier properties of films to oxygen and water vapor at 30% relative humidity (RH) and 23 °C.

Films	O_2_ Permeability Coefficient P × 10^17^ (kg·m·m^−2^·s^−1^·Pa^−1^)	Water Vapor Permeability Coefficient P × 10^14^ (kg·m·m^−2^·s^−1^·Pa^−1^)
PLA	2.2 ± 0.2 ^a^	6.6 ± 0.8 ^a^
PLA-*g*-TPCS	2.0 ± 0.1 ^a^	6.6 ± 0.3 ^a^
PLA-GRH	1.4 ± 0.1 ^b^	6.1 ± 0.1 ^a^
PLA-*g*-TPCS-GRH	0.9 ± 0.4 ^c^	6.1 ± 0.7 ^a^

Note: Within columns, values followed by a different letter are significantly different at *p* ≤ 0.05 (Tukey’s test).

## Supplementary Materials

The XRD characterization, color, opacity, and electrical resistivity of the films are available online at www.mdpi.com/2073-4360/10/1/95/s1, Table S1: *T_g_*, *T_cc_*, *T_m_*, and *X_c_* from the first heating cycle of DSC; Table S2: Values of optical properties of films produced by TSE-CF; Table S3: Resistivity of film samples; Figure S1: SEM images of graphene nanoplatelets powder used as reinforcement for production of the nanocomposites; Figure S2: SEM images of film samples before and after tensile test, evaluated in cross direction (CD) and machine direction (MD); Figure S3: XRD patterns obtained after tensile testing of film samples produced by twin-screw extrusion–cast-film extrusion; Figure S4: Picture of film samples produced by twin-screw extrusion followed by cast-film extrusion.
