# Supplementary material for: Toughening of Poly(lactic acid) and Thermoplastic Cassava Starch Reactive Blends Using Graphene Nanoplatelets
Source: Polymers (Basel). 2018 Jan 19;10(1):95. doi: 10.3390/polym10010095 (PMC6415146; doi:10.3390/polym10010095)
Supplement: Supplementary file 1 [file polymers-10-00095-s001.pdf]

Supporting information for: Toughening of Poly(lactic acid) and Thermoplastic Cassava Starch Reactive Blends Using Graphene Nanoplatelets

Anibal Bher [1,2,3], Ilke Uysal Unalan [1,4], Rafael Auras [1,*], Maria Rubino [1] and Carlos E. Schvezov [3]

[1] School of Packaging, Michigan State University, East Lansing, MI 48824, USA; anibalbher@gmail.com (A.B.); iuysalunalan@gmail.com (I.U.U.); mariar@msu.edu (M.R.)
[2] Instituto Sabato, UNSAM-CNEA, San Martin, Buenos Aires 1650, Argentina
[3] Instituto de Materiales de Misiones (IMAM), CONICET-UNaM, Posadas, Misiones 3300, Argentina; schvezov@gmail.com
[4] Department of Food Engineering, Faculty of Engineering, İzmir University of Economics, İzmir 35330, Turkey
* Correspondence: aurasraf@msu.edu; Tel.: +1-517-432-3254

SEM IMAGES OF GRAPHENE NANOPLATELETS USED AS A NANOFILLER 2
TENSILE TEST EVALUATED IN CROSS (CD) AND MACHINE (MD) DIRECTION 2
X-RAY DIFFRACTION (XRD) 3
DSC OF THE FILMS FOR THE FIRST HEATING CYCLE. 3
COLOR OF FILMS AND OPACITY 3
ELECTRICAL RESISTIVITY 4
REFERENCES 5

SEM images of graphene nanoplatelets used as a nanofiller

Samples were mounted on aluminum stubs using high vacuum carbon tabs (SPI Supplies, West Chester, PA, US). Samples were examined in a JEOL 7500F (field emission emitter) scanning electron microscope (JEOL Ltd., Tokyo, Japan) at various magnifications at 5 kV.

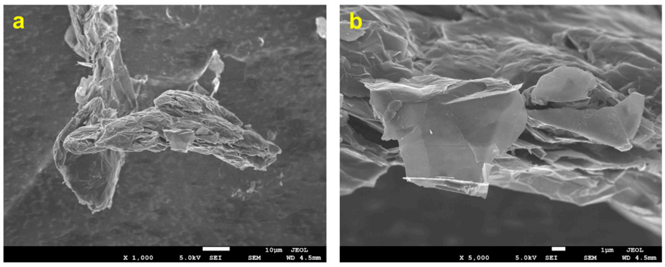

Figure S1. SEM images of graphene nanoplatelets powder used as reinforcement for production of the nanocomposites. (**a**) GRH (x1000), (**b**) GRH (x5000)

Tensile test evaluated in cross (CD) and machine (MD) direction

Tensile test method and SEM characterization are described in the Materials and Methods section of the paper.

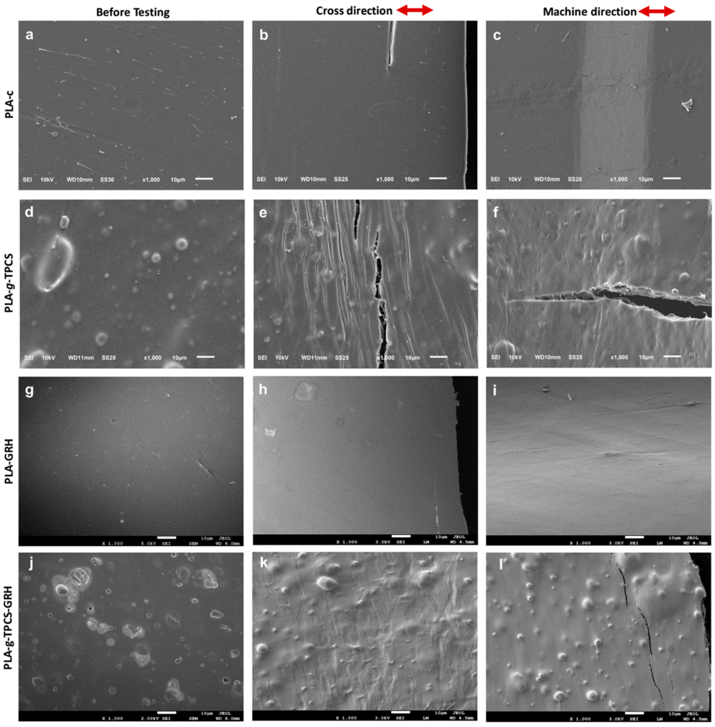

Figure S2. SEM images of film samples before and after tensile test, evaluated in cross direction (CD) and machine direction (MD): (**a**) PLA-c, (**b**) PLA-c (CD), (**c**) PLA-c (MD), (**d**) PLA-*g*-TPCS, (**e**) PLA-*g*-TPCS (CD), (**f**) PLA-*g*-TPCS (MD), (**g**) PLA-GRH, (**h**) PLA-GRH (CD), (**i**) PLA-GRH (MD), (**j**) PLA-*g*-TPCS-GRH, (**k**) PLA-*g*-TPCS-GRH (CD), (**l**) PLA-*g*-TPCS-GRH (MD).

X-ray diffraction (XRD)

XRD patterns of the cast films were recorded using a Bruker D8 Advance diffractometer (Bruker AXS, Madison, WI, US). Tests were performed under ambient temperature using an X-ray generator voltage of 40 kV and a current of 40 mA. The scan angle was from 5° to 40° with an increment of 0.010°·s^{-1}. The XRD instrument has a Cu tube with a wavelength of 1.4518 Å, and an anode of Cu with a Kα1 of 1.54060 Å, Kα2 of 1.54440 Å, and Kβ of 1.39224 Å. Three samples of each specimen were tested.

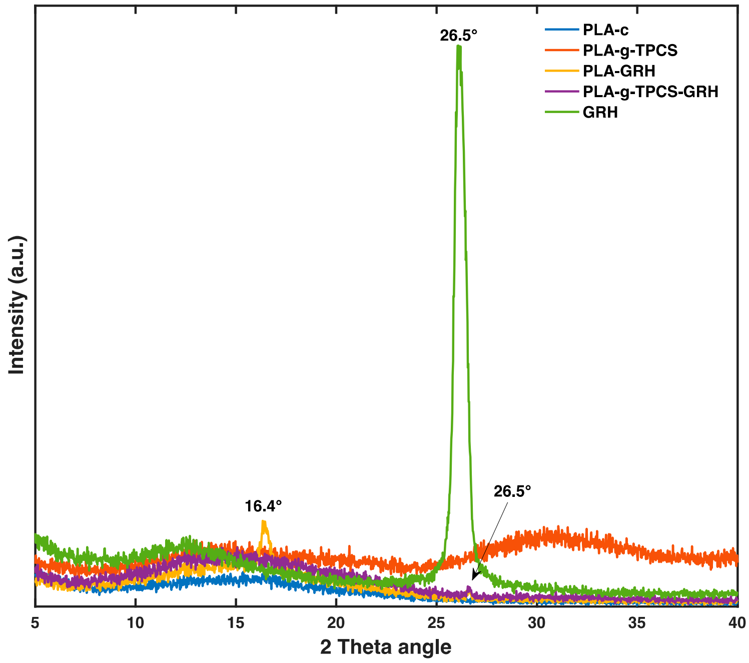

Figure S3. XRD patterns obtained after tensile testing of film samples produced by twin-screw extrusion–cast-film extrusion.

DSC of the films for the first heating cycle.

Table S1. T_g, T_{cc}, T_m, X_c from the first heating cycle of DSC

Films	T_g, °C	T_{cc}, °C	T_m, °C	X_c, %
PLA	60.9 ± 0.7^{a}	92.0 ± 3.7^{a}	151.0 ± 0.6^{a}	7.8 ± 6.0^{ab}
PLA-*g*-TPCS	54.0 ± 2.9^{b}	107.4 ± 0.9^{b}	143.4 ± 0.2^{b}	5.7 ± 3.0^{ab}
PLA-GRH	58.9 ± 1.0^{a}	85.9 ± 3.5^{a}	151.6 ± 0.2^{a}	1.7 ± 1.0^{a}
PLA-*g*-TPCS-GRH	56.7 ± 0.5^{ab}	101.9 ± 0.6^{b}	142.5 ± 0.4^{b}	12.0 ± 0.7^{b}

Note: Within columns, values followed by a different letter are significantly different at $p \leq 0.05$ (Tukey's test).

Color of films and opacity

The color of the films produced by twin-screw extrusion–cast-film extrusion (TSE-CF) were measured with the CIE L*a*b* system using a HunterLab LabScan XE spectrophotometer (Hunter

Associates Laboratory, Reston, VA, US). The tristimulus values XYZ were measured with the same equipment for determination of the yellowness index (YI).

Three specimens of each sample were measured. The opacity (*Op*) of the films was evaluated by measuring the absorbance at 550 nm (A_{550}), as described by Bao et al. [1]. The *Op* was calculated using equation [1], where *x* is the film thickness (expressed in mm).

$$Op = \frac{A_{550}}{x} \quad (1)$$

Table S2 shows the results of the tests.

Table S2. Values of optical properties of films produced by TSE-CF.

Films	L*	a*	b*	ΔE*	YI	%T, 300 nm	%T, 600 nm	*Op*
PLA	92.1±0.1[a]	-1.0±0.0[a]	1.0±0.0[a]	92.1±0.1[a]	8.4±0.0[a]	81.0±2.5[a]	88.2±1.4[a]	2.5±0.3[a]
PLA-*g*-TPCS	92.7±0.0[b]	-1.1±0.0[b]	1.4±0.0[b]	92.7±0.0[b]	9.1±0.0[b]	16.7±0.8[b]	21.3±0.6[b]	12.8±1.7[b]
PLA-GRH	90.8±0.1[c]	-0.9±0.0[c]	1.1±0.0[c]	90.8±0.1[c]	8.7±0.0[c]	80.5±1.5[a]	85.9±2.2[a]	2.6±0.3[a]
PLA-*g*-TPCS-GRH	90.6±0.1[c]	-1.0±0.0[d]	1.4±0.0[d]	90.6±0.1[c]	9.3±0.0[d]	15.6±1.7[b]	20.4±1.6[b]	14.9±0.1[b]

Note: Within columns, values followed by a different letter are significantly different at $p \leq 0.05$ (Tukey's test).

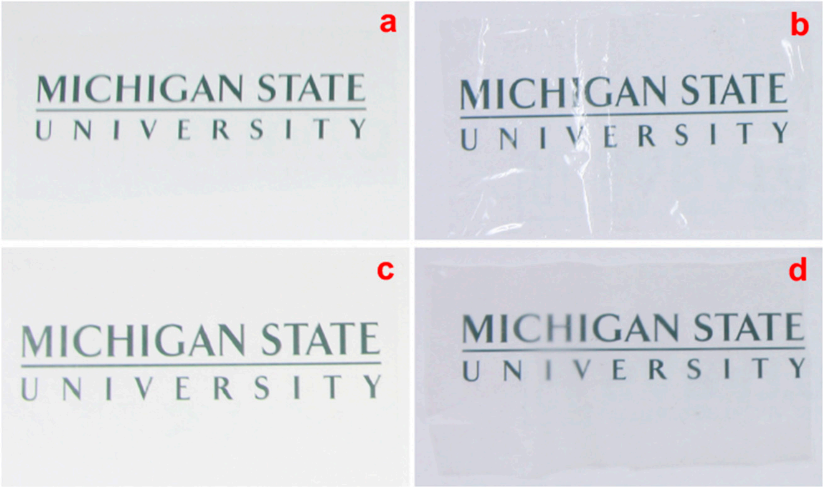

Figure S4. Picture of film samples produced by twin-screw extrusion followed by cast-film extrusion: (**a**) PLA-c, (**b**) PLA-GRH, (**c**) PLA-*g*-TPCS, (**d**) PLA-*g*-TPCS-GRH.

Electrical Resistivity

Electrical resistivity was evaluated using a FAS2™ Femtostat (Gamry Instruments, Warminster, PA, US). Film sample area was 6.3 cm^2; three specimens were measured for each type of film. Table S3 shows the results. A piece of copper tape was added to each side of the sample and connected to two terminals of the Potentiostat. Values of impedance, area and thickness of each sample were used to calculate the resistivity of each type of film.

Table S3. Resistivity of film samples.

Films	Resistivity ($\times 10^{12}$) (Ohm × m)
PLA-c	4.9 ± 0.1[a]
PLA-*g*-TPCS	5.4 ± 3.0[a]
PLA-GRH	1.9 ± 0.3[b]
PLA-*g*-TPCS-GRH	2.3 ± 0.5[b]

Note: Values followed by a different letter are significantly different at $p \leq 0.05$ (Tukey's test).

Reference

1. Bao, S.; Xu, S.; Wang, Z. Antioxidant activity and properties of gelatin films incorporated with tea polyphenol-loaded chitosan nanoparticles. *J. Sci. Food Agric.* **2009**, *89*, 2692–2700, doi:10.1002/jsfa.3775.
